# Exosome-mediated regulation of inflammatory pathway during respiratory viral disease

**DOI:** 10.1186/s12985-024-02297-y

**Published:** 2024-01-25

**Authors:** Hamidreza Gheitasi, Mohammad Sabbaghian, Ali Akbar Shekarchi, Amir Ali Mirmazhary, Vahdat Poortahmasebi

**Affiliations:** 1 Department of Bacteriology and Virology, Faculty of Medicine, Tabriz University of Medical Sciences, Tabriz, Iran; 2 Research Center for Clinical Virology, Tehran University of Medical Sciences, Tehran, Iran; 3 Department of Pathology, Faculty of Medicine, Tabriz University of Medical Sciences, Tabriz, Iran

**Keywords:** Exosomes, Viral infections, Respiratory diseases, Respiratory viruses

## Abstract

Viruses have developed many mechanisms by which they can stimulate or inhibit inflammation and cause various diseases, including viral respiratory diseases that kill many people every year. One of the mechanisms that viruses use to induce or inhibit inflammation is exosomes. Exosomes are small membrane nanovesicles (30–150 nm) released from cells that contain proteins, DNA, and coding and non-coding RNA species. They are a group of extracellular vesicles that cells can take up to produce and mediate communication. Intercellular effect exosomes can deliver a broad confine of biological molecules, containing nucleic acids, proteins, and lipids, to the target cell, where they can convey therapeutic or pathogenic consequences through the modulation of inflammation and immune processes. Recent research has shown that exosomes can deliver entire virus genomes or virions to distant target cells, then the delivered viruses can escape the immune system and infect cells. Adenoviruses, orthomyxoviruses, paramyxoviruses, respiratory syncytial viruses, picornaviruses, coronaviruses, and rhinoviruses are mostly related to respiratory diseases. In this article, we will first discuss the current knowledge of exosomes. We will learn about the relationship between exosomes and viral infections, and We mention the inflammations caused by viruses in the airways, the role of exosomes in them, and finally, we examine the relationship between the viruses as mentioned earlier, and the regulation of inflammatory pathways that play a role in causing the disease.

## Introduction

The respiratory system includes a set of organs of the human body that perform gas exchange between the human body and the outside air, organs such as the nose, pharynx, larynx, trachea, bronchi, lungs, etc. are among the organs. Which are widely used in the human respiratory system and are among the vital human organs [1]. Respiratory diseases including chronic obstructive pulmonary disease (COPD), asthma and obstructive sleep apnea (OSA), and acute conditions such as pneumonia have a high prevalence in the world population, and numerous reports show their gradual increase and have health and economic consequences. It has attracted the attention of many doctors and researchers [2]. Therefore, it is essential to identify and control the factors involved in respiratory diseases. Considering that various antiviral drugs such as oseltamivir and zanamivir have been proposed for respiratory diseases caused by the influenza virus, but the main problem here is that there is no clinically approved antiviral drug for common respiratory viruses, and there is also a practical vaccine does not have [3, 4]. Mutual recognition between viral respiratory infections and host responses and their associated molecular mechanisms has recently been established [5]. For example, during virus replication, infected cells may secrete cytokines that alter the local inflammation. Inflammation in the airways creates an anti-viral state, called type 1 inflammation, which can ultimately lead to the virus being removed [6, 7].

Microorganisms play a significant function in respiratory disease, for example, viruses and bacteria can cause respiratory diseases [8]. Respiratory syncytial viruses, influenza, parainfluenza, rhinoviruses, bocaviruses, and viruses of the coronavirus can cause respiratory diseases [9–14]. These viruses can cause respiratory problems in different ways; for example, they can transfer their DNA or RNA to exosomes [15].

Since viruses are tiny organisms with parasitic life, research has found a close relationship between viruses and exosomes [16]. Exosomes are phospholipid nanovesicles with cup-shaped morphology and diameters between 30 and 150 nm, found in blood, urine, and almost all eukaryotic fluids and cell culture media, and can be isolated using it [17]. Recent research has shown that exosomes act in two ways during viral infections. They can deliver the viral genome to the target cell or they can change the physiology of the target cell to facilitate infection [18].

In general, exosomes show an attractive field of research that promises a wide range of applications in medicine, and investigating the relationship between viruses and infections caused by them with blisters can be very helpful in exosome-based treatments and diagnostics. In this review, we do our best to provide an overview of the effects of respiratory infectious viruses and exosomes.

## Biogenesis, composition, and function of exosomes

**Fig. 1:**
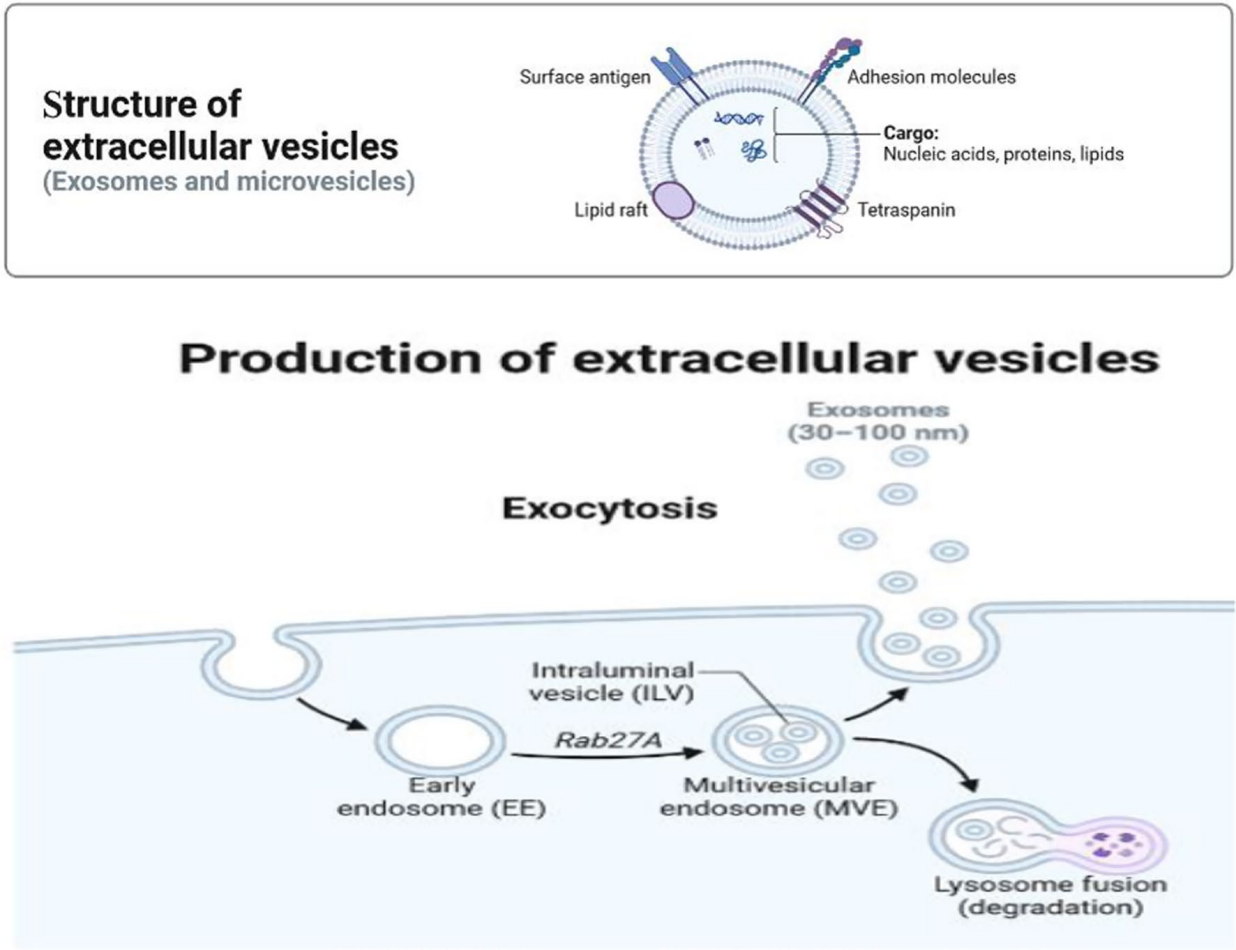
Schematic representation of exosome biogenesis and secretion. Internalization happens by endocytosis involving membrane proteins and lipid complexes. Then, endocytotic vesicles are transported to early endosomes and fuse to form late endosomes/multivesicular bodies (MVB). MVBs could fuse with the cellular membrane to release exosomes or fuse with lysosomes for degradation [29]. Created with BioRender.com

The term exosome was first used for extracellular vesicles (EVs) in 1981. In 1983, research showed that exosomes derived from reticulocytes can fuse with the plasma membrane and release their contents through exocytosis [19, 20]. EV is a general term for particles that are naturally removed from the cell and are bound by a lipid bilayer and cannot replicate, i.e. they do not contain a functional nucleus [21]. As mentioned, exosomes are particles between 30 and 150 nm that are secreted by most cells, and they are small extracellular vesicles that are derived from endosomes. Before being released into the external environment, they are formed and stored inside multivesicular bodies (MVBs) [22], as seen in Fig. 1. The different proteins and lipids that exist in the membrane of exosomes are caused by the phospholipid membrane that exosomes have acquired from the parental cells [23]. Phosphatidylcholine, phosphatidylethanolamines, phosphatidylinositol, phosphatidylserine, and sphingomyelin are present in the exosome membrane [24]. The high stability of exosomes in body fluids and at different pH depends on the composition and levels of these lipid molecules. This is due to the presence of high levels of sphingomyelin and phosphatidylinositol in their membranes [25]. Exosomes can be secreted from most cells in both physiological and pathological conditions, and by transferring vital genetic materials such as miRNA, mRNA, and DNA, as well as proteins and microorganisms such as viruses, they play an essential role in cellular communication and epigenetic regulation. As a result, they can be used to diagnose diseases based on exosomes and treatment methods [26, 27]. In culture, exosomes are also released from several cells, including B cells, dendritic cells (DCs), T cells, mast cells, and epithelial cells [28].

During biogenesis, the early exosomes formed by the endosomal system mature into late endosomes; during, this process, the contents of the endosomes construction due to the action of the v-ATPase booster pump, as well as the replacement of late endosome markers Rab 7 and -9. Rab. with mannose 6-phosphate receptor instead of 5-Rab [30]. After the formation of mature late endosomes, intraluminal vesicles (ILVs) are formed and accumulate in their lumen. There are two mechanisms for forming ILVs: endosomal sorting complex required for transport (ESCRT)-dependent and ESCRT-independent [30].

*ESCRT-related mechanism*: several mechanisms and molecules are involved in the formation of ILV, and the known ESCRT-related tool is the most ubiquitin-related [31]. ESCRT machines with SNARE producers and their effectors such as RAB GTPase play a prominent role in this process [32]. ESCRT consists of four complexes, ESCRT -0, ESCRT -I, ESCRT-II, and ESCRT-III, each having related functions (VPS4, Tsg101, and ALIX—classified lipid domains. ESCRT-I and ESCRT-II cause membrane deformation to form a stable membrane neck, and the use of the Vps4 complex in ESCRT-III causes vesicle neck cleavage and the separation of the ESCRT-III complex [33].

*Non-ESCRT-dependent mechanism*: Research shows that exosome biogenesis can have an EST-independent pathway, for example, showing that 4 ESCRT complexes are silenced. Intraluminal vesicles can still form in multivesicular bodies [34]. Non-ESCRT-dependent mechanism: research shows that exosome biogenesis can have an EST-independent pathway, for example, showing that 4 ESCRT complexes are silenced. Intraluminal vesicles can still form in multivesicular bodies [35]. Generally, non-ESCRT-dependent mechanisms can be investigated with two categories: lipid-mediated biogenesis and tetraspanin-mediated biogenesis.

*Lipid-Mediated Biogenesis*: Exosomes are enriched with cholesterol, sphingolipids, and ceramide. Neutral sphingomyelinase two and phospholipase D2 converts sphingomyelin to ceramide and phosphatidylcholine to phosphatidic acid (PA), respectively [36]. Ceramide and PA then form a cone-like structure that eventually leads to the formation of ILVs released as EVs [37].

*Tetraspanin-mediated biogenesis*: One of the ESCRT-independent regulators of exosome biogenesis that are highly enriched within exosomes and have been explicitly used as exosome biomarkers over the years is the tetraspanin family, of which CD9 is specifically used as an exosome marker [38].

The composition of exosomes depends on various factors, and it can also partially depend on the type of cell and is affected by different cell states; it, can also depend on its biogenesis, and it can also be determined by biological processes such as reverse budding of the plasma membrane [39]. According to the origin and biogenesis of exosomes, the composition of exosomes includes three major types of biomolecules: (a) proteins, such as proteins involved in membrane transport and fusion, heat shock proteins, Alix proteins, TSG101 and lipid-related proteins and phospholipases. (b) Lipids: lipids such as ceramides, cholesterol, long chain glycerophospholipids and sphingolipids and types of RNAs including mRNA, miRNA and other non-coding RNAs, tRNA and rRNA [40–42].

Exosome has a wide range of functions, the most important of which is the exchange of information and transfer of materials between cells. Exosomes communicate with cells by three main mechanisms: either they bind to the receptor of the target cell, or they combine directly with the membrane of the target cell, or they enter the target cell by endocytosis, which endocytosis can be clathrin-dependent or clathrin-independent. To occur, exosomes are heterogeneous in terms of size and content are released by different cell types and can regulate the biological activity of target cells by transferring proteins, lipids, and nucleic acids, for example, they are involved in various biological processes such as angiogenesis, antigen presentation, apoptosis, and inflammation play a role. Furthermore, research has demonstrated a strong correlation between illnesses and the molecular constituents present within exosomes [43–47].

## Role of exosome in the regulation of inflammation

In the last decade, studies reported that exosomes generated by various cell types have promising biological activity in not only promoting cell proliferation, angiogenesis, cancer cell metastasis, and invasion, but also reprogramming metabolic processes, maintaining physiological homeostasis, regulating inflammation, and modulating the immune evasion, etc. [48, 49]. This evidence sheds light on the potential therapeutic effects of exosomes for different chronic inflammatory diseases and tumors [50]. As summarized in Table 1, exosomes can play dual pro-inflammatory and anti-inflammatory roles during inflammation.

**Table 1:** Regulation of inflammation by exosome cargo

Exosome derived from	Disease	Mechanism	Pathway	Effect	References
Monocytes	Tumor cell	Exosomes derived from monocytes secrete various inflammatory cytokines, including IL6, IL-1β, IL-8, and TNF-α	STAT3 and NFκB	Pro-inflammatory effects	[51]
Macrophages	Breast and stomach tumor	Exosomes derived from macrophages in breast and stomach tumors stimulate the production of inflammatory cytokines, including GCSF, IL-6, IL-8, IL1β, CCL2, and TNF-α	NF-κB	Pro-inflammatory effects	[52]
Dendritic cells	Tumor cell	Induction of IL6 and Secretion of TGFβ	STAT3	Pro-inflammatory effects	[51, 53]
Myeloid-derived suppressor cells (MDSC)	Tumor cell	Induction of the proinflammatory cytokine Cox 2 and an increase in inflammatory cytokines such as IL-6, TNF-α, VEGF, CCL2	STAT3	Pro-inflammatory effects	[54]
Natural killer cells	Tumor	Inhibition of NK cell activation mediated by IL-2	By inducing Smad phosphorylation, it can disrupt cytotoxicity and reduce NKG2D receptor expression	Anti-inflammatory effects	[55]
Regulatory T cells (Treg)	Tumor	Expansion phosphorylation of relevant transcription factors IL-10 and TGF- β1	immunosuppression	Anti-inflammatory effects	[56]
Macrophages	_	Decreased expression of IL-6	Decreased expression of IL-6 It can also act as a negative regulator in the JAK/STAT pathway	Anti-inflammatory effects	[57, 58]

Today, inflammation is considered the leading cause of many respiratory diseases, which is associated with a high rate of complications and mortality [59]. Many blood vessels in the lung can cause the release of membrane mucins from exosomes derived from lung endothelial cells, which facilitates the innate defense of the airway [60]. As mentioned, nowadays, exosomes have received a lot of attention in various inflammatory lung diseases such as COPD, asthma, acute lung injury (ALI), and COVID-19. In the meantime, COPD is obtained from the damage of endothelial cells and epithelial cells along with epithelial-mesenchymal transition (EMT) in the lung parenchyma [61, 62]. The secretion of exosomes from lung endothelial cells significantly increases with exposure to smoke and infection in the lungs of COPD subjects, which leads to increased production of IL-8, followed by continuous damage and inflammation in the lung tissue [63]. After injury has occurred, the production of large numbers of exosomes derived from lung endothelial cells in the bronchoalveolar lavage fluid (BALF) can alter the function of receptor cells, which can predict the level of COPD injury [64]. In research by Lee et al., they indicated that alveolar macrophages and alveolar epithelial cells type I are the primary sources of exosomes in BALF of ARDS and ALI [65]. The production of more exosomes, as well as the increase of eosinophils in the airways, can improve asthma in most cases [66, 67]. COVID-19 is another cause of severe lung inflammation. Based on the analysis, exosomes derived from COVID-19 patients contain some molecules, such as complement subunits C1r and C1s, which can stimulate inflammatory responses [68]. Exosomes may be used in the production of antiviral vaccines as well as pharmaceutical platforms, which are due to the intercellular communication activity and the distinct structure of the two lipid layers of exosomes. Also, the development of lung cancer can also be one of the cases in which exosomes are used in. They play a role in the way that exosomes secreted by the tumor can affect angiogenesis, EMT, etc. [69–71]. All the above shows the high importance of exosomes and shows that exosomes can have a dual role that can be very useful for therapeutic work.

## Regulation of exosome biogenesis by viral proteins

### Virus regulates exosome production

Millions of people worldwide are affected by microbial diseases such as bacterial, parasitic, fungal, and viral diseases. In recent years, the role of exosomes has been much noticed, and the discovery of their relationship with viruses has progressed a lot [72, 73]. According to previous studies, it has been found that viruses can hijack the path of exosomes to facilitate the germination, accumulation, or release of the virus, and these hijacked exosomes help the virus escape from the immune system and spread in the body [74]. As mentioned, exosomes can act as viral carriers and directly contribute to virus replication; the first evidence to understand this issue came when researchers realized the importance of ESCRT components during viral capsid packaging and the maturation of several enveloped viruses [17]. Research has proven that viruses can regulate the biogenesis of exosomes, which play an essential role in virus and pathogen transmission. For example, the nef protein of HIV mediates the hijacking of exosome biogenesis. It directs proteins for integration into exosomes, or during infection with respiratory syncytial virus (RSV), it causes the cell to secrete more exosomes and change exosome components that are in N, F, G proteins of RSV virus are involved in this. Also, during hepatitis C virus infection, the viral genome can enter ILVs and later be secreted in exosomes. In the case of Epstein-Barr virus (EBV), exosomes may contain viral proteins that cause infection, or herpes simplex virus-1 (HSV-1)-derived exosomes may contain microRNAs that produce host-associated proteins. Virus-induced exosomes modulate antiviral immunity [72, 74–77].

Based on recent research, it has been found that exosome production from cells can be increased by exposure to viruses [78]. In a way, the number of late endosomes/MVBs could be enhanced by viral protein expression in different cells, such as human T lymphocytes [79], CIITA, HeLa, and Mel JuSo cells [80], resulting in raised release of ILVs. These results support the involvement of viral protein in exosome biogenesis in different cell types, such as astrocytes [81], CD4 + T cells, and U937 cells [82]. Considering the discovery of the viral protein domain and amino acid sequence responsible for stimulation of vesicle release [83], it has been found that N, G, and F proteins of respiratory syncytial virus (RSV) are involved in the secretion of more exosomes and changes in exosome components during infection [84]. According to the research of Wu and his colleagues, it was found that infection with enterovirus A71 (EV-A71) increases the secretion of exosomes both in vitro and in vivo [85]. It also increases the production of exosomes from macrophages infected by HIV-1 [86]. In general, some viruses can increase the production of exosomes and use them for their benefit by increasing the production of exosomes.

## Virus regulates exosome composition

Also, viral infection changes the exosomal proteome [87]. As mentioned, viral proteins have a substantial impact on changing the exosome’s composition. For instance, the incorporation of the Nef protein of human immunodeficiency virus (HIV) into the exosomes derived from HIV-infected cells [88], as well as Nef-transformed T cells [84, 85], stimulates the release of Nef from cells [89] and cell death of bystander CD4 + T lymphocytes. Of note, these T lymphocytes are essential and involved in the AIDS progression [89] and derive inflammation mediated by destroying cholesterol metabolism and elevating lipid rafts in bystander cells [90]. In addition, increased expression of ADP-ribosyl cyclase 1 (CD38) by exosomes released by HIV-infected T-lymphocytes occurs, which decreases annexin A5 (ANXA5) and L-lactate dehydrogenase B (LDHB) chain levels, which The above interact with Tat and P24, HIV viral proteins, and affect the cellular processes of proliferation and apoptosis [87]. In a research conducted in 2023, it was found that the exosomes of HBV-infected hepatocytes activate stellate cells, which can aggravate liver fibrosis [91].

Despite all the above information, the specific roles, and mechanisms of exosomal sorting of these proteins have not been fully discovered during viral infection and require more extensive research in this field.

## Regulation of inflammation by respiratory viruses through exosomes

### Respiratory syncytial viruses

In 1956, the respiratory syncytial virus (RSV) was discovered. Over the past years, researchers realized that it could cause lower respiratory tract disease at all ages and is the leading cause of death in children under five years of age, for which there is no vaccine. RSV is one of the RNA viruses that, according to statistics presented in 2019, causes 3.6 million hospital beds and causes the death of about 101,400 children aged 0–60 months [92–95]. RSV encodes eleven separate proteins, including structural proteins such as membrane envelope glycoproteins (F and G), two non-structural proteins 1 and 2 (NS1 and NS2), and matrix proteins (M), which are considered crucial pathogenic agents to stimulate airway hyperreactivity (AHR), such as Th2-type overexpression, cytokines immune disorder, and inflammatory disequilibrium [96–98]. RSV infection can affect the airway epithelium, polarized and differentiated cells release chemokines such as IL-8 and CCL5 and cause inflammatory cells to invade infected tissues [99]. Chemokines removed from the respiratory tract of RSV-infected children such as β-chemokines CCL2 (monocyte chemoattractant protein-1 [MCP-1]), CCL3 (macrophage inflammatory protein-1 [MIP-1α]), CCL11 (eotaxin), and CCL5 in the RSV-infected respiratory tract has been shown to not only predict disease severity, possibly by enhancing inflammation in response to RSV [100]. Reportedly, the RSV-infected cells could aggravate the release rates of exosomes carrying the RSV N protein, attachment G protein, fusion F protein, and a wide range of RNAs (e.g., mRNA, small noncoding-RNAs, ribosomal RNA segments, and RSV RNA) [101]. Chahar et al. reported that RSV-infected cells produced exosomes with a significant potential for enhanced release of the chemokines, IP-10, RANTES, and MCP-1, in monocytes compared to control cell exosomes. Similar to monocytes, RSV exosomes in comparison to mock exosomes enhanced the secretion of RANTES, IP-10, and TNF-α in the A549 cells, indicative of RSV exosomes ability in the generation of biological signals to alter innate immunity and inflammation during RSV infection [101]. Also, RSV can induce inflammation in different ways, and exosome is one of these ways; for, example, according to research on exosomes isolated from A549 cells infected with RSV in the laboratory, it was shown that the secretion of RANTES, Il-10, and TNF-α increases significantly and can induce inflammation [101]. Identifying these pathways can be very useful for treatment.

### Influenza virus

Influenza viruses, the members of the family *Orthomyxoviridae*, are negative-sense RNA viruses classified into A, B, C, and D types. Amongst, only type A and B cause seasonal epidemics in people. Influenza A virus (IAV) is subtyped based on the two surface glycoproteins expression, neuraminidase (NA) and hemagglutinin (HA). Infection begins with the direct deposition of influenza particles into the epithelium or alveoli of the upper respiratory tract. The virus then binds to the cellular receptors via HA on its surface [102, 103]. According to the CDC, research on all common types of influenza between 2010 and 2022 showed that children under four years are more likely to be infected with almost all pathogenic influenza types than other age groups. Each year, severe influenza infection affects about 3–5 million people, of whom nearly 250,000–500,000 give in to the disease [104]. It has been reported that mice infected with influenza virus have various microRNAs (miRNAs), such as miR-483-3p, in their BALF exosomes. It has been hypothesized that miR-483p may boost the innate immunity in MLE-12 epithelial cells rather than other lung epithelial cells due to the miR-483-3p potential in enhanced activation of the transcription factors NF-kB and IRF3 in MLE-12 cells [105]. Based on this, miR-483-3p could provide body protection against the flu. Also, one of the critical receptors used by the influenza virus to bind to the target cell is sialic acid, related to a2,3 and a2,6, which are expressed by exosomes released in the airways. According to these ways, it competes with the sialylated cell surface receptor to which HA binds and is harmful to subsequent infections. In this way, it is clear that influenza virus infectivity can be neutralized by airway exosomes [106]. Also, BALF exosomes infected with the virus have been found to produce IL-6, MCP-1, and TNF and can induce inflammation [106]. To use exosomes as therapeutic or vaccine platforms, it is helpful to identify the different pathways by which exosomes impose their effects.

### Parainfluenza virus

Human parainfluenza virus (HPIV), belonging to the family paramyxoviridae, is an enveloped, single-stranded, negative-sense RNA virus that can infect various host species. HPIV has four types [1, 107, 3] and two subtypes (4a and 4b) with different clinical and epidemiological characteristics [108]. HPIV is vital because there is no vaccine available for it to prevent the infection. Infections with parainfluenza viruses may result in various illnesses, such as conjunctivitis, pharyngitis, tracheobronchitis, otitis media, croup, and pneumonia. In children under five years, HPIV is the second leading reason for acute respiratory illness causing hospitalization after RSV [109]. HPIV infection increases the secretion of exosomes containing a massive load of viral proteins and RNA which can be transferred to other cells, causing productive infection and promoting viral replication. It has been shown that exosomes derived from HPIV-infected cells contain a different range of RNA species, including piRNA and miRNA. Autophagy inhibited by HPIV exosomes has also been demonstrated in other studies. The miR-126-3 p_2 was an essential regulatory factor in this process [110]. Probably, autophagy inhibition is one of the reasons for developing efficient exosome-mediated replication of HPIV infection. Also, miRNAs delivered to recipient cells through exosomes subsequently cause gene silencing, which has different effects depending on the cell type and stage [111]. Overall, the knowledge about inflammation caused by exosome and parainfluenza is minimal and needs further investigation.

### Rhinoviruses

Rhinoviruses are linear single-stranded RNA viruses that belong to the picornaviridae family and belong to the enterovirus genus [112]. So far, more than 150 serotypes of rhinoviruses have been identified, which primarily cause mild and self-limiting diseases in healthy people, but cause significant complications in people with lung diseases [113, 114]. More than half of upper respiratory tract infections include human rhinoviruses, which are known as colds, and eventually recover after a week [115]. Rhinoviruses cause inflammation in the airways by activating of interleukins such as IL1 [116]. Also, through Exosomal miRNAs (ExoMiRNAs), which are non-coding RNAs with a length of 18 to 25 nucleotides, they can affect the inflammation process [117]. According to research, it has been determined that exosomes derived from rhinoviruses that contain MiR155 can induce inflammation because miR-155 Th2-mediated eosinophilic inflammation is essential in the lung [118]. Also, various studies show the induction of inflammation by exosomes derived from rhinoviruses, which have different mechanisms; for, example they can induce inflammation by producing cytokines and chemokines such as CXCL8 [119].

### Coronaviruses

The coronaviruses family can cause common colds or acute and mild infections in the upper respiratory tract of both animals and humans. Coronavirus is a single-stranded (positive sense) RNA virus enclosed in an envelope with several protein molecules. The viral envelope consists of a lipid bilayer made of structural proteins: the membrane (M), coat (E), and spike (S). Coronaviruses are classified by the appearance of the corona or halo-like envelope glycoproteins, and chemical and replication characteristics. Coronaviruses enter the body through the respiratory system and attach to host cells using spike proteins. Then, they penetrate the cells and multiply and produce more viral particles. Upon internalization of the cells, an immune response is created to control the infection. However, an excessive immune response can lead to tissue damage and inflammation, especially in the respiratory system, called a cytokine storm [120, 121]. Coronaviruses can also affect other organs and methods of the body. The severity of the disease can range from mild respiratory signs to severe pneumonia and multi-organ failure [19]. The relationship between exosomes and coronavirus can be investigated in three ways: 1-entry of the virus, 2-immune modulation, and 3-detection potential [122–124].

Exosomes potentially facilitate the coronavirus entry into the target cells [15]. Coronaviruses, including SARS-CoV-2, modulate the host immune response. During coronavirus infection, exosomes carrying viral components or immunoregulatory molecules are released, which affect immune cell function and inflammatory responses [125]. Probably, exosomes derived from virus-infected cells contain various viral details, including viral protein and RNA [125]. As a diagnostic tool, there are many biomarkers for detecting coronaviruses through exosomes. According to the Fujita et al. study, these biomarkers can be classified into three categories as follows; 1-antiviral response-related biomarkers, 2-coagulation-related markers, and 3- liver damage-related biomarkers [126]. Much research has been conducted on coronaviruses, especially after the spread and epidemic of SARS-CoV-2, which shows interesting results. It has been found that these types of exosomes have stimulating effects on inflammation. Therefore, exosomes can be used in different ways to treat and prevent respiratory diseases.

During the tests on exosomes derived from lung macrophages, it was found that Nsp12 and Nsp13 in exosomes derived from them can lead to the activation of nuclear factor κB (NF-κB) and subsequent induction of an array of inflammatory cytokines [127]. Also, in another study, it was found that pro-inflammatory cytokines can be produced through the interaction of the NF-kB inflammatory signaling pathway with Fibrinogen-β (FGB) and Tenascin-C (TNC) and plasma exosomes can cause the transfer of FGB, which ultimately causes pro-inflammatory cytokine signals are removed in organ cells [128].

### Adenoviruses

Adenoviruses are a group of viruses that can cause various illnesses in humans. They can infect different tissues and are transmitted through close personal contact, respiratory droplets, or contaminated objects. Adenoviruses can provoke respiratory infections (common cold, sore throat, and pneumonia), gastrointestinal infections (diarrhea and vomiting), conjunctivitis (pink eye), and urinary tract infections [129, 130]. Most infected cases resolve independently, but severe conditions can occur in individuals with weakened immune systems. Prevention involves good hygiene practices and vaccination against certain adenovirus types. Consulting a healthcare professional is advised for diagnosis and treatment. The pathogenesis of adenovirus infection involves several steps, including entering the body through the respiratory or gastrointestinal tract, attaching to the specific receptors on host cells, releasing their DNA into the cell's nucleus followed by replication and transcription of viral genetic material, alongside synthesizing viral proteins [30]. From the infected cell, this is released to create new viral particles, these components assemble [131]. Adenoviruses can spread within the body and target various organs. The host immune system could be activated to eliminate the infection. Adenoviruses can evade immunity, resulting in prolonged or persistent disease, especially in individuals with weakened immune systems. The severity of adenovirus infections can vary, and different serotypes cause different clinical manifestations [132].

It has been revealed that adenoviruses use exosomes during their reproduction in the host cells. During infection, they can infect neighboring cells by release of exosomes from infected cells. These exosomes can contain viral components or factors that help the spread of the virus. Also, other studies have shown that adenoviruses can use exosomes to escape from the immune system [132].

 In addition, exosomes released by uninfected cells can contain antiviral agents that have the potential to inhibit virus replication or stimulate immune responses against the virus [133]. In the research conducted in the laboratory on A549 cells infected with adenovirus type 3, it was found that the exosomes derived from these infected cells significantly increased IL-1β, which caused the presence of the condition to become inflammatory [134]. In contrast to other studies, other studies indicate that DC/FasL-derived exosomes can be used clinically to treat inflammatory and autoimmune diseases [135].

In summary, adenoviruses can manipulate the exosome pathway during infection and stimulate the release of virus-induced exosomes from infected cells [16]. These exosomes can help spread the virus, modulate the immune system, and promote disease progression. In addition, exosomes derived from uninfected cells can influence adenovirus infection by transferring antiviral agents [15]. Exosomes from adenovirus-infected cells have been shown to both induce and inhibit inflammation.

## Conclusion and outlook

**Fig. 2:**
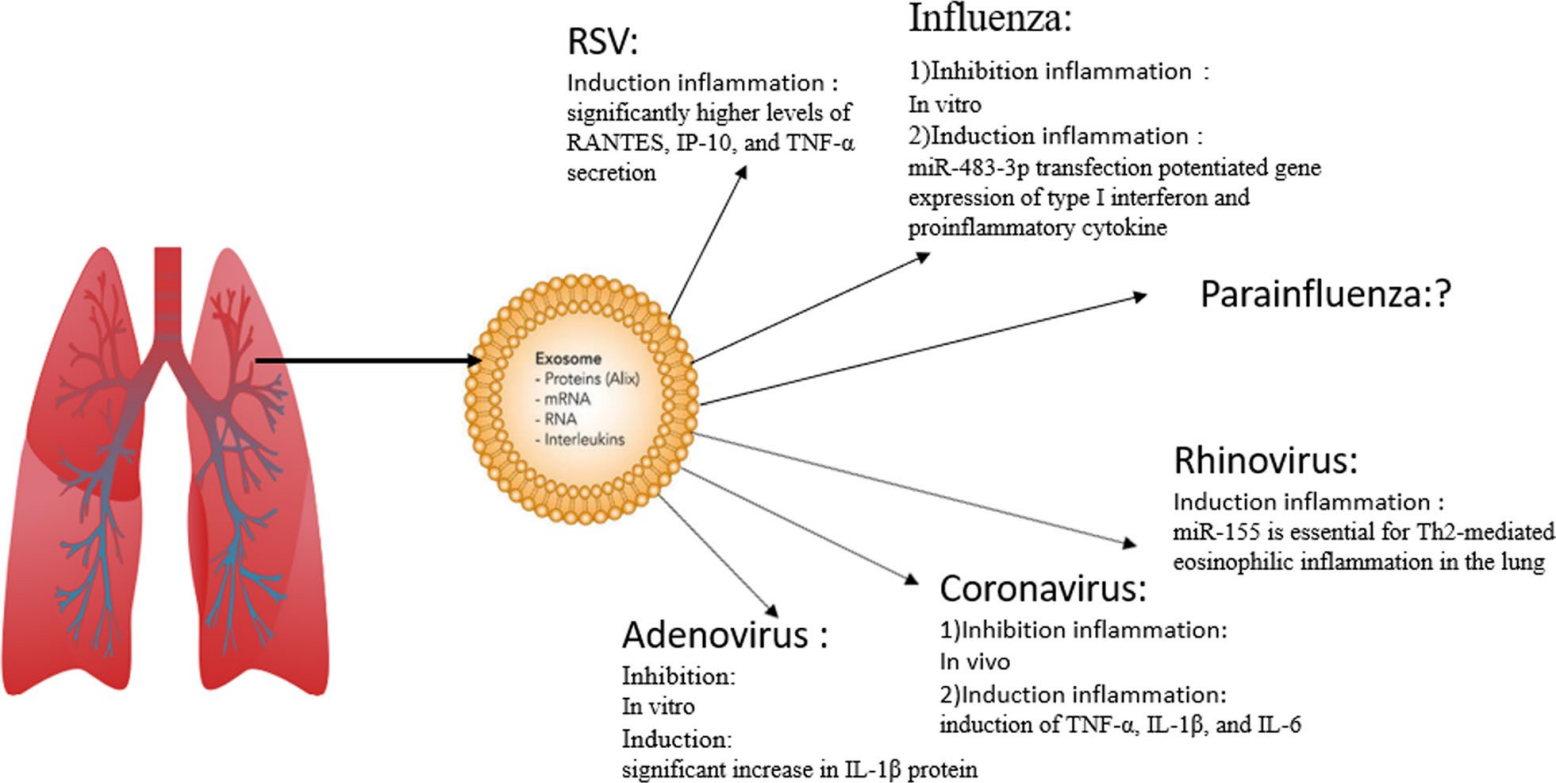
Schematic diagram of the role of exosomes in regulating inflammatory pathways in common viruses that cause respiratory disease

One of the things that plays an essential role in anti-virus immunity and virus promotion and is also an important vector for the transmission of viruses is exosome. The role of exosomes in the transmission of viruses and their role in inducing or inhibiting airway inflammation is critical, and as shown in Table 2 and Fig. 2, viruses that cause respiratory diseases can regulate inflammatory pathways through Exosomes that play a role in causing disease.

**Table 2:** Regulation of inflammatory pathways by viruses through exosomes

Virus	Exosome source	Exosome cargo	Sample (in vitro, in vivo)	Induction or inhibition of inflammation	Note	References
Respiratory syncytial viruses	RSV-infected A549 cells	–	In vitro (RSV-infected A549 cells)	Induction	Exosomes derived from RSV-infected A549 cells secrete significantly higher levels of RANTES, IP-10, and TNF-α, which activate the innate immune response and may through the release of pro-inflammatory cytokines and apoptosis in receptor cells have antiviral effects	[101]
Influenza	Respiratory epithelial cell lines	NP, NS1, M1, HA	In vitro (Respiratory epithelial cell lines)	Inhibition	Exosomes derived from respiratory epithelial cell lines infected with influenza virus showed an anti-viral response, which may inhibit inflammation in the early stages	[106]
Influenza	Bronchial alveolar lavage fluid (BALf) of influenza virus	–	In vitro (bronchial alveolar lavage fluid (BALf) of influenza virus)	Induction	Exosomes derived from bronchial alveolar lavage fluid (BALf) of influenza virus produce IL-6, MCP-1, and TNF, which are inflammatory cytokines and increase inflammation	[106]
Influenza	Macrophages, fibroblasts, T cell, and B cell lines	Nucleoprotein (NP) and non-structural protein (NS1)	In vitro (macrophages, fibroblasts, T cell, and B cell lines)	Induction	Levels of pro-inflammatory cytokines, such as IFN-γ, IL-1β, and CXCL8, were also elevated by the exosomes	[136]
Influenza	Bronchoalveolar lavage fluid (BALF)	miR-483-3p	In vitro (bronchoalveolar lavage fluid (BALF))	Induction	The transfer of miR-483-3p from bronchoalveolar lavage fluid (BALF) caused the expression of type I interferon and proinflammatory cytokine genes and strengthened their face	[137]
Influenza	Exosomes derived from H5N1-infected chickens	Viral proteins, NP and NS1	In vitro (exosomes derived from H5N1-infected chickens)	Induction	The presence of viral proteins such as NP and NS1 in exosomes derived from H5N1-infected chickens increases the level of pro-inflammatory cytokines such as IFN-γ, IL-1β, and IL-8	[119]
Influenza	Bronchoalveolar lavage fluid (BALF)	miR-483-3p, miR-374c-5p, miR-466i-5p, miR-203-3p	In vitro (bronchoalveolar lavage fluid (BALF))	Induction	miR-483-3p, miR-374c-5p, miR-466i-5p, miR-203-3p in exosomes derived from bronchoalveolar lavage fluid (BALF) significantly increased IFN-β expression, proinflammatory cytokine gene expression, and upregulates interferon-stimulated genes (ISGs), including IL6, CCL2, TNF-α, and SP110	[138]
Parainfluenza	Madin–Darby bovine kidney (MDBK) cells inoculated with CPIV3	miRNA 11	In vitro (Madin–Darby bovine kidney (MDBK) cells inoculated with CPIV3)	Unknown	These exosomes could transfer CPIV3 genetic materials to recipient cells to establish a productive infection and promote viral replication	[139]
Rhinoviruses	Primary bone marrow-derived DCs (BMDCs)	MiR155	in vitro and in vivo	Induction	miR-155 in exosomes derived from primary bone marrow-derived DCs (BMDCs) is essential for Th2-mediated eosinophilic inflammation in the lung and induces inflammatory conditions in the body	[118]
Rhinoviruses	RV-infected AECs	–	In vitro	Induction	Exosomes from RV-infected AECs yielded significant inflammatory cytokines/chemokines such as CXCL8, which induced an inflammatory state in the body	[140]
Rhinoviruses	Bronchoalveolar lavage fluid	–	In vitro	Induction	–	[141]
Coronaviruses	Lung macrophages	Nsp12 and Nsp13	In vitro	Induction	Nsp12 and Nsp13 in exosomes derived from lung macrophages lead to the activation of nuclear factor κB (NF-κB) and subsequent induction of an array of inflammatory cytokines	[127]
Coronaviruses	Patient plasma	Tenascin-C (TNC) and fibrinogen-β (FGB)	In vitro	Induction	Tenascin-C (TNC) and fibrinogen-β (FGB) induce the production of pro-inflammatory cytokines through interaction with the NF-κB inflammatory signaling pathway. FGB is transported via plasma exosomes and potentially induces pro-inflammatory cytokine signals in distant organ cells	[128]
Coronaviruses	A549 cell	Viral protein E, Nsp7, Nsp10, Nsp12, Nsp13, and slight protein M	In vitro	Induction	The RNA polymerase, Nsp12, alone inhibits tumor necrosis factor (TNF)-α and interleukin (IL)-6. Furthermore, a synergistic effect of Nsp12 working with Nsp13 was observed where, compared to Nsp12 alone, there was a significant induction of TNF-α, IL-1β, and IL-6, which are inflammatory cytokines. In contrast, M protein, Nsp13 alone, or Nsp10 did not	[127]
Coronaviruses	Endothelial cells	–	In vitro	Induction	Exosomes derived from endothelial cells infected with coronaviruses lead to NLRP3 inflammasome activation in endothelial cells of distant organs, which ultimately leads to IL-1β secretion and inflammatory response	[128]
Adenoviruses	Bone marrow-derived dendritic cells (DCs)	Expressing viral IL-10	In vitro	Inhibition		[142]
Adenoviruses	Derived from FasL-expressing DC	–	In vitro	Inhibition	Mice are immunized with a specific antigen, keyhole limpet hemocyanin (KLH). Then a Th1-mediated inflammatory response is induced 10 to 14 days after immunization by injection of the particular antigen into the hind pads. Mice immunized with KLH 106 DC or 1 μg exosome received in one hind paw 12 h before KLH booster injection into both hind paws. Delivery of DC/FasL or DC/FasL-derived exosomes significantly reduced paw swelling not only in the treated paw but also in the contralateral paw. Untreated suppressed 24, 48, and 72 h after antigen injection. These results show that genetically modified DC-expressing FasL as well as DC/FasL-derived exosomes are equally effective in suppressing the DTH response not only in the treated paw, but they are also in the untreated opposite paw	[135]
Adenovirus	Infected A549	–	In vitro	Induction	A549-derived exosomes infected with adenoviruses significantly increased IL-1β protein, which induced inflammatory conditions in the body	[134]

## Data Availability

Not applicable.

## References

[1] Ding L, Qi H, Wang Y, Zhang Z, Liu Q, Guo C (2023). Recent advances in ginsenosides against respiratory diseases: therapeutic targets and potential mechanisms. Biomed Pharmacother.

[2] Turcani P, Skrickova J, Pavlik T, Janousova E, Orban M (2015). The prevalence of obstructive sleep apnea in patients hospitalized for COPD exacerbation. Biomed Papers.

[3] Chan-Zapata I, Borges-Argáez R, Ayora-Talavera G (2023). Quinones as promising compounds against respiratory viruses: a review. Molecules.

[4] Behl T, Rocchetti G, Chadha S, Zengin G, Bungau S, Kumar A (2021). Phytochemicals from plant foods as potential source of antiviral agents: an overview. Pharmaceuticals.

[5] Sparrer KMJ, Gack MU (2015). Intracellular detection of viral nucleic acids. Curr Opin Microbiol.

[6] Schoborg RV, Borel N (2014). Porcine epidemic diarrhea virus (PEDV) co-infection induced chlamydial persistence/stress does not require viral replication. Front Cell Infect Microbiol.

[7] Singh A, Bisht P, Bhattacharya S, Guchhait P (2020). Role of platelet cytokines in dengue virus infection. Front Cell Infect Microbiol.

[8] Ożarowski M, Karpiński TM (2023). The effects of propolis on viral respiratory diseases. Molecules.

[9] Venkatesan P (2022). Nirsevimab: a promising therapy for RSV. The Lancet Microbe.

[10] Chotpitayasunondh T, Fischer TK, Heraud JM, Hurt AC, Monto AS, Osterhaus A (2021). Influenza and COVID-19: what does co-existence mean?. Influenza Other Respir Viruses.

[11] Cordisco M, Lucente MS, Sposato A, Cardone R, Pellegrini F, Franchini D (2022). Canine parainfluenza virus infection in a dog with acute respiratory disease. Vet Sci.

[12] Ortega H, Nickle D, Carter L (2021). Rhinovirus and asthma: challenges and opportunities. Rev Med Virol.

[13] He Y, Zhang J, Feng J, Mei Z, Qian L, Huang Q (2021). Viral spectrum and clinical features in adult inpatients with community-acquired pneumonia and respiratory viral infection. Chin Gen Pract.

[14] Hu B, Guo H, Zhou P, Shi Z-L (2021). Characteristics of SARS-CoV-2 and COVID-19. Nat Rev Microbiol.

[15] Chaudhari P, Ghate V, Nampoothiri M, Lewis S (2022). Multifunctional role of exosomes in viral diseases: from transmission to diagnosis and therapy. Cell Signal.

[16] Peng Y, Yang Y, Li Y, Shi T, Luan Y, Yin C (2023). Exosome and virus infection. Front Immunol.

[17] Shi Y, Du L, Lv D, Li Y, Zhang Z, Huang X (2021). Emerging role and therapeutic application of exosome in hepatitis virus infection and associated diseases. J Gastroenterol.

[18] Fu Y, Zi R, Xiong S. Infection by exosome-carried Coxsackievirus B3 induces immune escape resulting in an aggravated pathogenesis. Microbes Infect. 2023:105148. 10.1016/j.micinf.2023.105148 37156458

[19] Li P, Kaslan M, Lee SH, Yao J, Gao Z (2017). Progress in exosome isolation techniques. Theranostics.

[20] Gao M, Gao W, Papadimitriou J, Zhang C, Gao J, Zheng M (2018). Exosomes—the enigmatic regulators of bone homeostasis. Bone Res.

[21] Théry C, Witwer KW, Aikawa E, Alcaraz MJ, Anderson JD, Andriantsitohaina R (2018). Minimal information for studies of extracellular vesicles 2018 (MISEV2018): a position statement of the International Society for Extracellular Vesicles and update of the MISEV2014 guidelines. J Extracell Vesicles.

[22] Jeppesen DK, Fenix AM, Franklin JL, Higginbotham JN, Zhang Q, Zimmerman LJ (2019). Reassessment of exosome composition. Cell.

[23] Jiang Y, Wang F, Wang K, Zhong Y, Wei X, Wang Q (2022). Engineered exosomes: a promising drug delivery strategy for brain diseases. Curr Med Chem.

[24] Ding D, He X, Agarry IE, Wang Y, Zhou F, Li Y (2023). Profile of human milk phospholipids at different lactation stages with UPLC/Q-TOF-MS: characterization, distribution, and differences. J Agric Food Chem.

[25] Paskeh MDA, Entezari M, Mirzaei S, Zabolian A, Saleki H, Naghdi MJ (2022). Emerging role of exosomes in cancer progression and tumor microenvironment remodeling. J Hematol Oncol.

[26] Yang D, Zhang W, Zhang H, Zhang F, Chen L, Ma L (2020). Progress, opportunity, and perspective on exosome isolation-efforts for efficient exosome-based theranostics. Theranostics.

[27] Sims B, Gu L, Krendelchtchikov A, Matthews QL (2014). Neural stem cell-derived exosomes mediate viral entry. Int J Nanomed.

[28] Admyre C, Telemo E, Almqvist N, Lötvall J, Lahesmaa R, Scheynius A (2008). Exosomes–nanovesicles with possible roles in allergic inflammation. Allergy.

[29] Than UTT, Guanzon D, Leavesley D, Parker T (2017). Association of extracellular membrane vesicles with cutaneous wound healing. Int J Mol Sci.

[30] Burkova EE, Sedykh SE, Nevinsky GA (2021). Human placenta exosomes: biogenesis, isolation, composition, and prospects for use in diagnostics. Int J Mol Sci.

[31] Tschuschke M, Kocherova I, Bryja A, Mozdziak P, Angelova Volponi A, Janowicz K (2020). Inclusion biogenesis, methods of isolation and clinical application of human cellular exosomes. J Clin Med.

[32] Gurung S, Perocheau D, Touramanidou L, Baruteau J (2021). The exosome journey: from biogenesis to uptake and intracellular signalling. Cell Commun Signal.

[33] Yue B, Yang H, Wang J, Ru W, Wu J, Huang Y (2020). Exosome biogenesis, secretion and function of exosomal miRNAs in skeletal muscle myogenesis. Cell Prolif.

[34] Jia G, Sowers JR (2020). Targeting endothelial exosomes for the prevention of cardiovascular disease. Biochim et Biophys Acta (BBA)-Mol Basis Dis..

[35] Anakor E, Le Gall L, Dumonceaux J, Duddy WJ, Duguez S (2021). Exosomes in ageing and motor neurone disease: biogenesis, uptake mechanisms, modifications in disease and uses in the development of biomarkers and therapeutics. Cells.

[36] Ghossoub R, Lembo F, Rubio A, Gaillard CB, Bouchet J, Vitale N (2014). Syntenin-ALIX exosome biogenesis and budding into multivesicular bodies are controlled by ARF6 and PLD2. Nat Commun.

[37] McMahon HT, Boucrot E (2015). Membrane curvature at a glance. J Cell Sci.

[38] Charrin S, Jouannet S, Boucheix C, Rubinstein E (2014). Tetraspanins at a glance. J Cell Sci.

[39] Palmulli R, van Niel G (2018). To be or not to be… secreted as exosomes, a balance finely tuned by the mechanisms of biogenesis. Essays Biochem.

[40] Zhao R, Zhao T, He Z, Cai R, Pang W (2021). Composition, isolation, identification and function of adipose tissue-derived exosomes. Adipocyte.

[41] Lai JJ, Chau ZL, Chen SY, Hill JJ, Korpany KV, Liang NW (2022). Exosome processing and characterization approaches for research and technology development. Adv Sci.

[42] Huang J, Xiong J, Yang L, Zhang J, Sun S, Liang Y (2021). Cell-free exosome-laden scaffolds for tissue repair. Nanoscale.

[43] Tian Y, Cheng C, Wei Y, Yang F, Li G (2022). The role of exosomes in inflammatory diseases and tumor-related inflammation. Cells.

[44] Masyuk AI, Masyuk TV, LaRusso NF (2013). Exosomes in the pathogenesis, diagnostics and therapeutics of liver diseases. J Hepatol.

[45] Mao W, Wang K, Wu Z, Xu B, Chen M (2021). Current status of research on exosomes in general, and for the diagnosis and treatment of kidney cancer in particular. J Exp Clin Cancer Res.

[46] Beatriz M, Vilaça R, Lopes C (2021). Exosomes: innocent bystanders or critical culprits in neurodegenerative diseases. Front Cell Dev Biol.

[47] Jiao Y, Xu P, Shi H, Chen D, Shi H (2021). Advances on liver cell-derived exosomes in liver diseases. J Cell Mol Med.

[48] Efthymiou G, Saint A, Ruff M, Rekad Z, Ciais D, Van Obberghen-Schilling E (2020). Shaping up the tumor microenvironment with cellular fibronectin. Front Oncol.

[49] Santiago-Sánchez GS, Pita-Grisanti V, Quiñones-Díaz B, Gumpper K, Cruz-Monserrate Z, Vivas-Mejía PE (2020). Biological functions and therapeutic potential of Lipocalin 2 in cancer. Int J Mol Sci.

[50] Chan BD, Wong WY, Lee MM, Cho WC, Yee BK, Kwan YW (2019). Exosomes in inflammation and inflammatory disease. Proteomics.

[51] Othman N, Jamal R, Abu N (2019). Cancer-derived exosomes as effectors of key inflammation-related players. Front Immunol.

[52] Chow A, Zhou W, Liu L, Fong M, Champer J, Van Haute D (2014). Macrophage immunomodulation by breast cancer-derived exosomes requires Toll-like receptor 2-mediated activation of NF-ĸB. Sci Rep.

[53] Valenti R, Huber V, Filipazzi P, Pilla L, Sovena G, Villa A (2006). Human tumor-released microvesicles promote the differentiation of myeloid cells with transforming growth factor-β–mediated suppressive activity on T lymphocytes. Can Res.

[54] Chalmin F, Ladoire S, Mignot G, Vincent J, Bruchard M, Remy-Martin J-P (2010). Membrane-associated Hsp72 from tumor-derived exosomes mediates STAT3-dependent immunosuppressive function of mouse and human myeloid-derived suppressor cells. J Clin Investig.

[55] Szczepanski MJ, Szajnik M, Welsh A, Whiteside TL, Boyiadzis M (2011). Blast-derived microvesicles in sera from patients with acute myeloid leukemia suppress natural killer cell function via membrane-associated transforming growth factor-β1. Haematologica.

[56] Szajnik M, Czystowska M, Szczepanski MJ, Mandapathil M, Whiteside TL (2010). Tumor-derived microvesicles induce, expand and up-regulate biological activities of human regulatory T cells (Treg). PLoS ONE.

[57] Yoshimura A, Naka T, Kubo M (2007). SOCS proteins, cytokine signalling and immune regulation. Nat Rev Immunol.

[58] Li D, Jia H, Zhang H, Lv M, Liu J, Zhang Y (2012). TLR4 signaling induces the release of microparticles by tumor cells that regulate inflammatory cytokine IL-6 of macrophages via microRNA let-7b. Oncoimmunology.

[59] Calderón MA (2023). Cardiopulmonary axis and cardiovascular mortality in patients with COPD. SEMERGEN.

[60] Kesimer M, Gupta R (2015). Physical characterization and profiling of airway epithelial derived exosomes using light scattering. Methods (San Diego Calif).

[61] De SK (2023). Novel 4-chloro-N-phenyl benzamide derivatives as p38α mitogenactivated protein kinase inhibitors for treating cancer, COVID-19, and other diseases. Recent Pat Anti-Cancer Drug Discov.

[62] Nishioka M, Venkatesan N, Dessalle K, Mogas A, Kyoh S, Lin TY (2015). Fibroblast-epithelial cell interactions drive epithelial-mesenchymal transition differently in cells from normal and COPD patients. Respir Res.

[63] Chang Y, Al-Alwan L, Alshakfa S, Audusseau S, Mogas AK, Chouiali F (2014). Upregulation of IL-17A/F from human lung tissue explants with cigarette smoke exposure: implications for COPD. Respir Res.

[64] Liu Z, Yan J, Tong L, Liu S, Zhang Y (2022). The role of exosomes from BALF in lung disease. J Cell Physiol.

[65] Lee H, Zhang D, Laskin DL, Jin Y (1950). Functional evidence of pulmonary extracellular vesicles in infectious and noninfectious lung inflammation. J Immunol (Baltim Md: 1950).

[66] Gauvreau GM, El-Gammal AI, O'Byrne PM (2015). Allergen-induced airway responses. Eur Respir J.

[67] Esnault S, Kelly EA, Johnson SH, DeLain LP, Haedt MJ, Noll AL (2019). Matrix metalloproteinase-9-dependent release of IL-1β by human eosinophils. Mediators Inflamm.

[68] Kapferer-Seebacher I, Pepin M, Werner R, Aitman TJ, Nordgren A, Stoiber H (2016). Periodontal Ehlers-Danlos syndrome is caused by mutations in C1R and C1S, which encode subcomponents C1r and C1s of complement. Am J Hum Genet.

[69] Frydrychowicz M, Kolecka-Bednarczyk A, Madejczyk M, Yasar S, Dworacki G (2015). Exosomes—structure, biogenesis and biological role in non-small-cell lung cancer. Scand J Immunol.

[70] Wang B, Tan Z, Guan F (2019). Tumor-derived exosomes mediate the instability of cadherins and promote tumor progression. Int J Mol Sci.

[71] Xu K, Zhang C, Du T, Gabriel ANA, Wang X, Li X (2021). Progress of exosomes in the diagnosis and treatment of lung cancer. Biomed Pharmacother Biomed Pharmacother.

[72] Jia X, Yin Y, Chen Y, Mao L (2021). The role of viral proteins in the regulation of exosomes biogenesis. Front Cell Infect Microbiol.

[73] Saad MH, Badierah R, Redwan EM, El-Fakharany EM (2021). A comprehensive insight into the role of exosomes in viral infection: dual faces bearing different functions. Pharmaceutics.

[74] Vlachakis D, Mitsis Τ, Nicolaides N, Efthimiadou A, Giannakakis A, Bacopoulou F (2021). Functions, pathophysiology and current insights of exosomal endocrinology. Mol Med Rep.

[75] Dyball LE, Smales CM (2022). Exosomes: biogenesis, targeting, characterization and their potential as “Plug & Play” vaccine platforms. Biotechnol J.

[76] Mosquera-Heredia MI, Morales LC, Vidal OM, Barceló E, Silvera-Redondo C, Vélez JI (2021). Exosomes: potential disease biomarkers and new therapeutic targets. Biomedicines.

[77] Gurunathan S, Kang M-H, Kim J-H (2021). A comprehensive review on factors influences biogenesis, functions, therapeutic and clinical implications of exosomes. Int J Nanomed.

[78] Anderson MR, Kashanchi F, Jacobson S (2016). Exosomes in viral disease. Neurotherapeutics.

[79] Sanfridson A, Hester S, Doyle C (1997). Nef proteins encoded by human and simian immunodeficiency viruses induce the accumulation of endosomes and lysosomes in human T cells. Proc Natl Acad Sci USA.

[80] Stumptner-Cuvelette P, Jouve M, Helft J, Dugast M, Glouzman AS, Jooss K (2003). Human immunodeficiency virus-1 Nef expression induces intracellular accumulation of multivesicular bodies and major histocompatibility complex class II complexes: potential role of phosphatidylinositol 3-kinase. Mol Biol Cell.

[81] Pužar Dominkuš P, Ferdin J, Plemenitaš A, Peterlin BM, Lenassi M (2017). Nef is secreted in exosomes from Nef.GFP-expressing and HIV-1-infected human astrocytes. J Neurovirol.

[82] Aqil M, Naqvi AR, Mallik S, Bandyopadhyay S, Maulik U, Jameel S (2014). The HIV Nef protein modulates cellular and exosomal miRNA profiles in human monocytic cells. J Extracell Vesicles.

[83] Ali SA, Huang MB, Campbell PE, Roth WW, Campbell T, Khan M (2010). Genetic characterization of HIV type 1 Nef-induced vesicle secretion. AIDS Res Hum Retroviruses.

[84] Chahar HS, Corsello T, Kudlicki AS, Komaravelli N, Casola A (2018). Respiratory syncytial virus infection changes cargo composition of exosome released from airway epithelial cells. Sci Rep.

[85] Wu J, Zhao Y, Chen Q, Chen Y, Gu J, Mao L (2023). Enterovirus A71 promotes exosome secretion by the nonstructural protein 3A interacting with Rab27a. Microbiol Spectrum.

[86] Kadiu I, Narayanasamy P, Dash PK, Zhang W, Gendelman HE (2012). Biochemical and biologic characterization of exosomes and microvesicles as facilitators of HIV-1 infection in macrophages. J Immunol.

[87] Li M, Aliotta JM, Asara JM, Tucker L, Quesenberry P, Lally M (2012). Quantitative proteomic analysis of exosomes from HIV-1-infected lymphocytic cells. Proteomics.

[88] Muratori C, Cavallin LE, Krätzel K, Tinari A, De Milito A, Fais S (2009). Massive secretion by T cells is caused by HIV Nef in infected cells and by Nef transfer to bystander cells. Cell Host Microbe.

[89] Lenassi M, Cagney G, Liao M, Vaupotic T, Bartholomeeusen K, Cheng Y (2010). HIV Nef is secreted in exosomes and triggers apoptosis in bystander CD4+ T cells. Traffic.

[90] Mukhamedova N, Hoang A, Dragoljevic D, Dubrovsky L, Pushkarsky T, Low H (2019). Exosomes containing HIV protein Nef reorganize lipid rafts potentiating inflammatory response in bystander cells. PLoS Pathog.

[91] Yin M, Ding X, Yin S, Wang L, Zhang K, Chen Y, et al. Exosomes from hepatitis B virus‐infected hepatocytes activate hepatic stellate cells and aggravate liver fibrosis through the miR‐506‐3p/Nur77 pathway. J Biochem Mol Toxicol. 2023:e23432. 10.1002/jbt.23432 37352222

[92] Cao D, Gooneratne I, Mera C, Vy J, Royal M, Huang B (2023). Analysis of template variations on RNA synthesis by respiratory syncytial virus polymerase. Viruses.

[93] Wittenauer R, Pecenka C, Baral R (2023). Cost of childhood RSV management and cost-effectiveness of RSV interventions: a systematic review from a low-and middle-income country perspective. BMC Med.

[94] Falsey AR, Williams K, Gymnopoulou E, Bart S, Ervin J, Bastian AR (2023). Efficacy and safety of an Ad26. RSV. preF-RSV preF protein vaccine in older adults. N Engl J Med.

[95] Graham BS (2023). The journey to RSV vaccines—heralding an era of structure-based design. N Engl J Med.

[96] Caidi H, Miao C, Thornburg NJ, Tripp RA, Anderson LJ, Haynes LM (2018). Anti-respiratory syncytial virus (RSV) G monoclonal antibodies reduce lung inflammation and viral lung titers when delivered therapeutically in a BALB/c mouse model. Antiviral Res.

[97] Hu M, Bogoyevitch MA, Jans DA (2020). Impact of respiratory syncytial virus infection on host functions: implications for antiviral strategies. Physiol Rev.

[98] Walker JA, McKenzie AN (2018). TH2 cell development and function. Nat Rev Immunol.

[99] Linssen RSN, Sridhar A, Moreni G, van der Wel NN, van Woensel JBM, Wolthers KC (2022). Neutrophil extracellular traps do not induce injury and inflammation in well-differentiated RSV-infected airway epithelium. Cells.

[100] Lin HY, Chang KT, Hung CC, Kuo CH, Hwang SJ, Chen HC (2014). Effects of the mTOR inhibitor rapamycin on monocyte-secreted chemokines. BMC Immunol.

[101] Chahar HS, Corsello T, Kudlicki AS, Komaravelli N, Casola A (2018). Respiratory syncytial virus infection changes cargo composition of exosome released from airway epithelial cells. Sci Rep.

[102] Londrigan SL, Short KR, Ma J, Gillespie L, Rockman SP, Brooks AG, Reading PC (2015). Infection of mouse macrophages by seasonal influenza viruses can be restricted at the level of virus entry and at a late stage in the virus life cycle. ABSTRACT J Virol.

[103] Laporte M, Stevaert A, Raeymaekers V, Boogaerts T, Nehlmeier I, Chiu W, Benkheil M, Vanaudenaerde B, Pöhlmann S, Naesens L (2019). Hemagglutinin cleavability acid stability and temperature dependence optimize influenza B virus for replication in human airways. ABSTRACT J Virol.

[104] Gounder AP, Boon A (2019). Influenza pathogenesis: the effect of host factors on severity of disease. J Immunol.

[105] Maemura T, Fukuyama S, Kawaoka Y (2020). High levels of miR-483-3p are present in serum exosomes upon infection of mice with highly pathogenic avian influenza virus. Front Microbiol.

[106] Bedford JG, Infusini G, Dagley LF, Villalon-Letelier F, Zheng MZ, Bennett-Wood V (2020). Airway exosomes released during influenza virus infection serve as a key component of the antiviral innate immune response. Front Immunol.

[107] Allegra A, Murdaca G, Gammeri L, Ettari R, Gangemi S (2023). Alarmins and MicroRNAs, a new axis in the genesis of respiratory diseases: possible therapeutic implications. Int J Mol Sci.

[108] Welch M, Park J, Harmon K, Zhang J, Piñeyro P, Giménez-Lirola L (2021). Pathogenesis of a novel porcine parainfluenza virus type 1 isolate in conventional and colostrum deprived/caesarean derived pigs. Virology.

[109] Schomacker H, Schaap-Nutt A, Collins PL, Schmidt AC (2012). Pathogenesis of acute respiratory illness caused by human parainfluenza viruses. Curr Opin Virol.

[110] Tang F, Yang T-L (2018). MicroRNA-126 alleviates endothelial cells injury in atherosclerosis by restoring autophagic flux via inhibiting of PI3K/Akt/mTOR pathway. Biochem Biophys Res Commun.

[111] Pegtel DM, Cosmopoulos K, Thorley-Lawson DA, van Eijndhoven MA, Hopmans ES, Lindenberg JL (2010). Functional delivery of viral miRNAs via exosomes. Proc Natl Acad Sci.

[112] Real-Hohn A, Groznica M, Kontaxis G, Zhu R, Chaves OA, Vazquez L (2023). Stabilization of the quadruplex-forming G-rich sequences in the rhinovirus genome inhibits uncoating&mdash; role of Na+ and K+. Viruses.

[113] Coultas JA, Cafferkey J, Mallia P, Johnston SL. Experimental antiviral therapeutic studies for human rhinovirus infections. J Exp Pharmacol. 2021:645–59. 10.2147/JEP.S255211 PMC8277446 34276229

[114] McIntyre CL, Knowles NJ, Simmonds P (2013). Proposals for the classification of human rhinovirus species A B and C into genotypically assigned types. J General Virol.

[115] Blaas D, Fuchs R (2016). Mechanism of human rhinovirus infections. Mol Cell Pediatr.

[116] Montgomery ST, Frey DL, Mall MA, Stick SM, Kicic A, Arest CF (2020). Rhinovirus infection is associated with airway epithelial cell necrosis and inflammation via interleukin-1 in young children with cystic fibrosis. Front Immunol.

[117] Isaac R, Reis FCG, Ying W, Olefsky JM (2021). Exosomes as mediators of intercellular crosstalk in metabolism. Cell Metab.

[118] Gutierrez MJ, Gomez JL, Perez GF, Pancham K, Val S, Pillai DK (2016). Airway secretory microRNAome changes during rhinovirus infection in early childhood. PLoS ONE.

[119] Jantaratrirat S, Boonarkart C, Ruangrung K, Suptawiwat O, Auewarakul P (2018). Microparticle release from cell lines and its anti-influenza activity. Viral Immunol.

[120] Juckel D​, Desmarets L, Danneels A​, Rouillé Y, Dubuisson J, Belouzard S. MERS-CoV and SARS-CoV-2 membrane proteins are modified with polylactosamine chains. J General Virol. 2023;104(10). 10.1099/jgv.0.001900 10.1099/jgv.0.001900 37800895

[121] Pišlar A, Mitrović A, Sabotič J, Pečar U, Perišić FM, Tanja N, Senjor JE (2020). Kos J (2020) The role of cysteine peptidases in coronavirus cell entry and replication: The therapeutic potential of cathepsin inhibitors. PLOS Pathogens.

[122] Lanyu Z, Feilong H (2019). Emerging role of extracellular vesicles in lung injury and inflammation. Biomed Pharmacother.

[123] Martinez-Bravo M-J, Wahlund CJ, Qazi KR, Moulder R, Lukic A, Rådmark O (2017). Pulmonary sarcoidosis is associated with exosomal vitamin D-binding protein and inflammatory molecules. J Allergy Clin Immunol.

[124] Askenase PW (2020). COVID-19 therapy with mesenchymal stromal cells (MSC) and convalescent plasma must consider exosome involvement: do the exosomes in convalescent plasma antagonize the weak immune antibodies?. J Extracell Vesicles.

[125] Pesce E, Manfrini N, Cordiglieri C, Santi S, Bandera A, Gobbini A (2022). Exosomes recovered from the plasma of COVID-19 patients expose SARS-CoV-2 spike-derived fragments and contribute to the adaptive immune response. Front Immunol.

[126] Rosell A, Havervall S, Von Meijenfeldt F, Hisada Y, Aguilera K, Grover SP (2021). Patients with COVID-19 have elevated levels of circulating extracellular vesicle tissue factor activity that is associated with severity and mortality—brief report. Arterioscler Thromb Vasc Biol.

[127] Teng Y, Xu F, Zhang X, Mu J, Sayed M, Hu X (2021). Plant-derived exosomal microRNAs inhibit lung inflammation induced by exosomes SARS-CoV-2 Nsp12. Mol Ther.

[128] Sur S, Khatun M, Steele R, Isbell TS, Ray R, Ray RB (2021). Exosomes from COVID-19 patients carry tenascin-C and fibrinogen-β in triggering inflammatory signals in cells of distant organ. Int J Mol Sci.

[129] Shieh WJ (2022). Human adenovirus infections in pediatric population - An update on clinico–pathologic correlation. Biomed J.

[130] Stawicki SP, Jeanmonod R, Miller AC (2020). The 2019–2020 novel coronavirus (severe acute respiratory syndrome coronavirus 2) pandemic: A joint american college of academic international medicine-world academic council of emergency medicine multidisciplinary COVID-19 working group consensus paper. J Global Infect Dis.

[131] Zeng X, Carlin CR (2013). Host cell autophagy modulates early stages of adenovirus infections in airway epithelial cells. J Virol.

[132] Ipinmoroti AO, Matthews QL (2020). Extracellular vesicles: roles in human viral infections, immune-diagnostic, and therapeutic applications. Pathogens.

[133] Crenshaw BJ, Gu L, Sims B, Matthews QL (2018). Exosome biogenesis and biological function in response to viral infections. Open Virol J.

[134] Ipinmoroti AO, Crenshaw BJ, Pandit R, Kumar S, Sims B, Matthews QL. Human adenovirus serotype 3 infection modulates the biogenesis and composition of lung cell-derived extracellular vesicles. J Immunol Res. 2021;2021. 10.1155/2021/2958394 PMC8677401 34926703

[135] Kim SH, Bianco N, Menon R, Lechman ER, Shufesky WJ, Morelli AE (2006). Exosomes derived from genetically modified DC expressing FasL are anti-inflammatory and immunosuppressive. Mol Ther.

[136] Hong Y, Truong AD, Vu TH, Lee S, Heo J, Kang S (2022). Exosomes from H5N1 avian influenza virus-infected chickens regulate antiviral immune responses of chicken immune cells. Dev Comp Immunol.

[137] Maemura T, Fukuyama S, Sugita Y, Lopes TJS, Nakao T, Noda T (2018). Lung-derived exosomal miR-483-3p regulates the innate immune response to influenza virus infection. J Infect Dis.

[138] Zabrodskaya Y, Plotnikova M, Gavrilova N, Lozhkov A, Klotchenko S, Kiselev A (2022). Exosomes released by influenza-virus-infected cells carry factors capable of suppressing immune defense genes in Naïve cells. Viruses.

[139] Mao L, Liang P, Li W, Zhang S, Liu M, Yang L (2020). Exosomes promote caprine parainfluenza virus type 3 infection by inhibiting autophagy. J Gen Virol.

[140] Mills J (2018). Investigating the role of tenascin-C and extracellular vesicles in human rhinovirus induced exacerbations of asthma.

[141] Mills JT, Schwenzer A, Marsh EK, Edwards MR, Sabroe I, Midwood KS (2019). Airway epithelial cells generate pro-inflammatory tenascin-C and small extracellular vesicles in response to TLR3 stimuli and rhinovirus infection. Front Immunol.

[142] Kim S-H, Lechman ER, Bianco N, Menon R, Keravala A, Nash J (2005). Exosomes derived from IL-10-treated dendritic cells can suppress inflammation and collagen-induced arthritis 1. J Immunol.

